# Alcohol quantity and quality price elasticities: quantile regression estimates

**DOI:** 10.1007/s10198-018-1009-8

**Published:** 2018-10-01

**Authors:** Robert Pryce, Bruce Hollingsworth, Ian Walker

**Affiliations:** 1 School of Health and Related Research, University of Sheffield, 30 Regent Street, Sheffield, S1 4DA UK; 2 Division of Health Research, Lancaster University, Lancaster, LA1 4YG UK; 3 Economics Department, Lancaster University, Lancaster, LA1 4YG UK

**Keywords:** Alcohol demand, Quantile regression, Quality elasticity, D12, I18

## Abstract

Many people drink more than the recommended level of alcohol, with some drinking substantially more. There is evidence that suggests that this leads to large health and social costs, and price is often proposed as a tool for reducing consumption. This paper uses quantile regression methods to estimate the differential price (and income) elasticities across the drinking distribution. This is also done for on-premise (pubs, bars and clubs) and off-premise (supermarkets and shops) alcohol separately. In addition, we examine the extent to which drinkers respond to price changes by varying the ‘quality’ of the alcohol that they consume. We find that heavy drinkers are much less responsive to price in terms of quantity, but that they are more likely to substitute with cheaper products when the price of alcohol increases. The implication is that price-based policies may have little effect in reducing consumption amongst the heaviest drinkers, provided they can switch to lower quality alternatives.

## Introduction

UK household expenditure on alcohol in 2014 was over £20 billion or around 4% of GNP. Over 500 million litres of pure alcohol (equivalent to approximately 2 bottles of wine per-adult per week) was cleared by Her Majesty’s Revenue and Customs, generating over £9 billion in tax revenue [1]. The social cost of alcohol in the United Kingdom has been estimated at over £21 billion, of which £3.5 billion is attributed to health costs, £11 billion to crime, and £7 billion to lost productivity. The problem is not unique to the United Kingdom: according to World Health Organisation, alcohol is the second largest risk factor for disease and disability in Europe [2]. WHO advocates tax increase as one means of reducing consumption [2]. However, majority of the population (69% of men, 84% of women [3]) drink within the guidelines and any price-based policy, such as tax increase, would have a negative effect on these people, reducing the consumer surplus that they enjoy. Findings in the literature suggest a nonlinear relationship between alcohol consumption and overall mortality for both males and females [4]. This pattern is also found for specific health conditions including liver cirrhosis [5], oral and pharyngeal cancers [6], and stroke [7]. Whether this nonlinearity constitutes a J-curve is, however, a point of debate within the scientific literature (see, for example, Fillmore et al. [8] or Knott et al. [9]). One important parameter for policy makers, who might wish to reduce the overall consumption of alcohol, is the price elasticity of demand. However, if there is evidence of a nonlinear effect, then it might be possible to reduce harmful drinking without penalising moderate drinkers too greatly, providing the price elasticity was high for heavy drinkers and low for moderate drinkers. If that were true then taxation would reduce the harm on the heavy drinkers without imposing a large loss in consumer surplus for moderate users. Thus, it is particularly important to know how the price elasticity of demand varies across the distribution of drinking. While our prior is that heavy drinkers are likely to have a more inelastic demand than moderate drinkers, to the extent alcohol is addictive or habituating, there is little evidence to substantiate these priors. The theory of rational addiction [10] has been a major contribution to the literature but suggests that, for a given level of consumption, long-run price elasticities are lower than short-run ones. The usual empirical implementation of the theory is based on a very specific parameterisation that does not explicitly allow the slope of the demand curve to vary with consumption. However, we would expect addicts (rational, or otherwise) with linear demand to have higher levels of consumption at any price so implying a lower price elasticity.

One policy option to mitigate the problem of heavy drinking is minimum unit pricing, which sets a price floor for alcohol and so raises the price of low-priced alcohol. Since heavy drinkers typically purchase cheaper per unit alcoholic beverages (see, for example, Ludbrook et al. [11]), minimum unit pricing disproportionately increases the price for heavier drinkers than it does for light drinkers. Scotland introduced minimum unit pricing on alcohol in May 2018, with a floor price of 50 pence ($0.67; €0.57) per unit. Modelling by Brennan et al. [12] suggests that a 45 pence (US$0.60; €0.51) minimum unit price would affect the price of 12.5% of the units purchased by ‘moderate’ drinkers compared to 30.5% of the units purchased by ‘harmful’ drinkers^1^. The price elasticities in this modelling predict that a 45 pence minimum unit price would decrease consumption by 0.6% for moderate drinkers, compared to a decrease of 3.7% for harmful drinkers. However, the modelling is based on pseudo-panel estimates that impose a constant price elasticity across the drinking distribution^2^ [13]. If harmful drinkers were less price responsive than moderate drinkers, then the effects predicted in the modelling work will be incorrect. Since the marginal health and social harms are assumed to be increasing with consumption, the modelling work will thus overstate the health and social harm reduction of minimum unit pricing. The contribution of this paper is that it examines how the response to price varies across the drinking distribution using quantile regression methods. Moreover, it examines, for the first time, how quality substitution differs in response to price across the drinking distribution.

The contribution of this paper is that we provide estimates, using quantile regression methods for both quantity and quality, which show that the consumption of heavy drinkers is less responsive to price than that of moderate drinkers. Moreover, we also find that they are more likely to substitute with cheaper drinks when the price of alcohol increases. The implication, contrary to other influential works, is that price-based policies may have little effect in reducing consumption amongst the heaviest drinkers, at least when it is possible for them to switch to lower quality alternatives.

## Background literature

There is a large literature on the price elasticity of demand of alcohol. Two meta-analyses have been undertaken. Gallet [14] includes 132 studies, and reports a median price elasticity of demand of − 0.535, while Wagenaar et al. [15] includes many of the same studies and reports a mean price elasticity of − 0.44. The fact that the median is greater than the mean suggests that the distribution of elasticities found in the literature is negatively skewed. It should also be noted that the literature reviewed in the meta-analyses is varied, including studies looking at particular age groups (for example, adolescents).

Purshouse et al. [16] use the same underlying dataset as used in our own analysis to estimate price elasticities for use in policy simulation analysis. They estimate separate elasticities for moderate and harmful drinkers, and find that harmful drinkers are more responsive to price changes. They also estimate cross-price elasticities between low- and high-quality products within a beverage type, for example, low- and high-quality beer. These cross-price elasticities are very small, and significance levels are not provided. The method used does not account for the endogenous selection issue raised by Koenker and Hallock [17].

The seminal work on quantile regression in the context of alcohol demand was done by Manning et al. [18] which estimates the price elasticity of demand for alcohol in the United States. However, it uses only a single cross-section of data and a price index (ACCRA) which is the weighted average of three drinks (one beer, one whisky, and one wine). The variation in price comes only from across the cross-section and so is entirely driven by differences in the geographical location of consumers—so it is not possible to separately identify price effects from geographical effects. While, the identification strategy casts serious doubt on the interpretation of the estimated price elasticities, the results suggest a *U*-shaped relationship between conditional consumption decile and price elasticity, with the middle of the drinking distribution being most responsive to price changes relative to the tails of the distribution. Importantly for policy, they also find that the elasticity estimate for the top conditional decile, where the very heaviest drinkers get most weight, is not significantly different from zero.

Saffer et al. [19] use the National Longitudinal Study of Youth (NLSY) cohort study to estimate the response to price (and advertising) changes across the drinking distribution. The data are only concerned with those aged 18–29, so the results may not be generalisable to the whole population. The ACCRA price data is used. The authors use conditional quantile regression, and find no statistically significant relationship between price and consumption at any quantile and there was no statistical difference between estimates for any pair of deciles.

Byrnes et al. [20] used 3 years of Australian household cross-section survey data, and a double-hurdle quantile regression technique, to estimate the price elasticity of demand for alcohol. In contrast to Manning et al., the authors find that heavy drinkers are more responsive to price compared to lighter drinkers, and it is the lightest drinkers whose demand is perfectly inelastic. They also find a relatively high average price elasticity, close to − 1, which is much higher than the estimates found in the meta-analyses. The authors suggest that this finding could be specific to Australia and not generalisable to other countries. The authors also discuss the possibility of switching to cheaper alcohol products to mitigate price increase, but no analysis is carried out to test this possibility.

Gruenewald et al. [21] use time-series Swedish alcohol retail data from 1984 to 1994 to examine the impact of a change in the alcohol duty rates on alcohol quantity and quality demand. In 1992, the Swedish alcohol regulator, Systembolaget, changed the structure of duties such that beverages were taxed based on alcoholic strength rather than as a percentage of pre-tax price. The duty change led to a narrower distribution of prices for wine and spirits, but a wider distribution of prices for beer. The authors define quality by the relative price of the drink, and assign drinks into three categories—high, medium and low quality. This is done for three drink types—beer, wine and spirits—giving nine different types. However, the study uses only time-series data, with the dependent variable being monthly sales by drink type, giving 120 observations for each type. The price variable is a price index constructed from the unweighted average price for each of the nine drink types.

## Data

The data used in this paper come from 13 years of nationally representative, cross-sectional surveys: the UK Expenditure and Food Survey (EFS) 2001–2007, and its successor, the Living Costs and Food Survey 2008–2013. The surveys are a combination of a household interview and a 2-week expenditure diary. Detailed information on expenditure is recorded on alcoholic drinks, including information about the quantity (in millilitres) of alcoholic drinks purchased. Alcohol is recorded for 25 disaggregated premise type/drink type combinations—for example, on-trade/fortified wine. The consumption of drinks by type were converted into units of ethanol using the alcohol strengths reported in Purshouse et al. [16]. The diaries are recorded by individual expenditures within the household but we have no information on individual consumption, so this paper aggregates individual expenditures to the household level because of concerns regarding intra-household transfers.

We adopt a double log specification for demand and only households who purchased alcohol (68.4% of all households) are included in the study. The reason for zero expenditure is not known^3^ in our data and, in any event, it is not possible to use a simple Tobit specification when the dependent variable is in log form. The presumption in our double log specification is that there is no price that would turn a drinker into a non-drinker (and vice versa) and so non-drinkers simply have different preferences to drinkers.

We identify our price elasticities from cross-region and cross-time (in months) variation in prices that are derived from our microdata collapsed into region × time cells. Since our dataset is the same dataset that is used to construct the sub-indices of the retail prices index from observed expenditure and quantities, this is a natural way of defining prices. However, we do not include the lead and lag of consumption since our data is just pooled cross-sections. Implicitly, our specification could be reconciled with the conventional rational addiction specification by treating the omitted leads and lags as unobservables, and it deals with the effect of the resulting heteroskedasticity on standard errors by estimating the used robust methods.

Our survey data contains information on expenditure and quantity—so a “unit value” can be calculated by dividing expenditure by quantity. However, using unit values as the price would yield biased elasticity estimates since much of the variation in unit values would be due to variation in the ‘quality’ of alcohol consumed. That is, a difference in unit values across households would arise because of both differences in the true price and of differences in (endogenous) quality selection. If heavier drinkers have a taste for cheaper, lower ‘quality’ alcohol, then price elasticity estimates will be biased away from zero (i.e., will be more elastic).

For this reason, this study uses the month–region average price-per-unit of alcohol. Thus, our measure of price captures differential changes over time and across region. To create the index for a region, a mean price-per-unit is calculated for each household in that region by summing up alcohol expenditure and dividing by the total number of alcoholic units. The average of these is taken for cells defined by the 12 regions in the United Kingdom, and for each of the 153 months in the survey period.^4^ Note that since we include region fixed effects and month fixed effects, our “price” variable is defined as the regional × time average, and this definition is immune from the usual endogeneity arguments. We assume that prices change in response to changes in production costs, as well as tax changes over time.

**Fig. 1:**
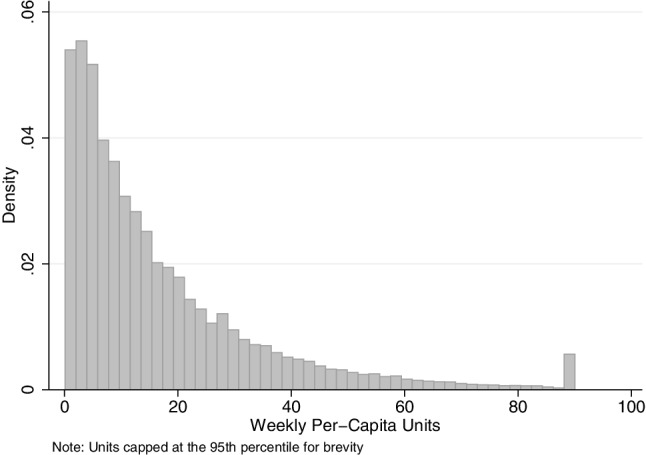
Distribution of per capita expenditure on units of alcohol

Figure 1 shows the distribution of per-adult alcohol consumption in the data. The distribution is truncated at the 99th percentile to compress the x axis, but this is not done in our statistical analysis. The distribution has a long right-hand tail, but it is clear that while the majority of households drink moderately.

**Fig. 2:**
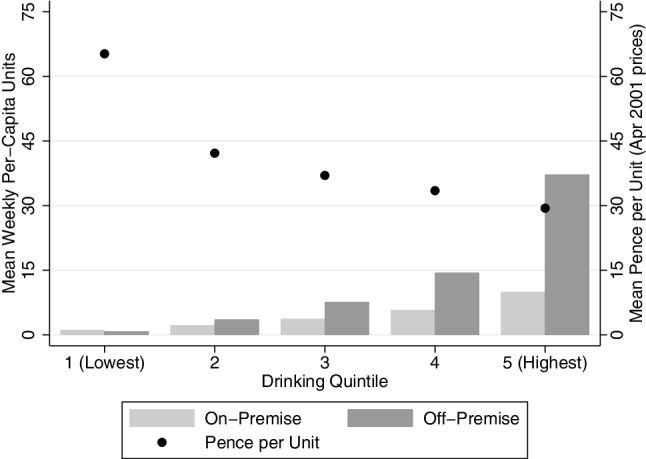
Differences in price and drinking location by quintile

Figure 2 illustrates that the heaviest drinking quintile drink proportionately more of their units in the off-trade (i.e., at home). The absolute levels of consumption are still higher in the on-trade for the heaviest drinking quintile compared to the lightest drinking quintile. Figure 2 also shows the mean price per unit paid by each drinking quintile. As reported in the literature, the heaviest drinkers tend to pay less per unit of alcohol.

Further summary statistics are provided in Table 1. It shows that there is little difference in characteristics across drinking quintiles, although the heaviest quintile spend more on average, and have slightly fewer adults and children in the household.

**Table 1:** Summary statistics

Variable	All respondents	Drinking quintile (adjusted for number of adults)				
		1 (lightest)	2	3	4	5 (heaviest)
Weekly per-adult units	17.25	1.9	5.77	11.32	20.18	47.15
On-premise units	4.55	1.12	2.24	3.69	5.75	9.95
Off-premise units	12.71	0.78	3.53	7.63	14.43	37.2
Per-adult total weekly expenditure (£)	142.67	128.74	130.68	138.13	147.67	168.2
Number of adults	1.92	2.02	1.94	1.94	1.92	1.76
Number of children	0.58	0.59	0.58	0.6	0.59	0.52
Age of oldest household member	51.72	52.92	51.79	51.27	50.81	51.82

## Methods

In contrast to Purshouse et al. [16] who do not account for Koenker and Hallock [17] in terms of estimating the demand equation separately for light, moderate and heavy drinkers, this paper uses conditional quantile regression, which seeks to minimise the (weighted) absolute deviations, in contrast to OLS which minimises the unweighted squared deviations. The method uses all the data simultaneously and simply varies the weights given to each observation across deciles. Conditional quantile regression essentially provides the difference between the conditional distributions at the given quantile. As a robustness check, we also use unconditional quantile regression. This method, developed by Firpo et al. [22], attempts to better estimate the marginal effects across the distribution using the recentered influence function (RIF). Policymakers are likely to be more interested in the unconditional distribution than the conditional distribution, because the policy is naturally concerned with those who are unconditionally heavy drinkers, rather than those who are heavy drinkers, by virtue of their observed characteristics. A discussion of the differences between unconditional and conditional quantile regression is provided by Borah and Basu [23]. In any case, there are no substantive differences between the estimates generated by conditional quantile regression compared to unconditional quantile regression.

To estimate the price elasticity of quantity demanded, we use the double-log model

1 $$\ln {Q_{hrt}}=\alpha +~{\beta _1}\ln {P_{rt}}+{\eta _1}\ln {Y_{hrt}}+{\delta _1}\ln {A_{hrt}}+\varvec{\gamma}_{1}^{'}{{\varvec{Z}}_{{\varvec{h}}{\varvec{r}}{\varvec{t}}}}+~{\upsilon _{hrt}}$$

which predicts the units $Q$ consumed by household $h$ in region $r$ at time $t$ as a function of: price $P$, which varies only by *r* and *t;* total per capita weekly expenditure $Y$; the number of adults in the household $A$; and other control variables ${{\varvec{Z}}_{{\varvec{h}}{\varvec{r}}{\varvec{t}}}}$ which include the number of children in the household, the age of the oldest household member, a linear time trend, and monthly and regional fixed effects. ${\upsilon _{hrt}}$ is an error term which is assumed to be normally distributed with mean zero. Because the double-log model is used, ${\beta _1}$ can be interpreted as the price elasticity of quantity demanded of alcohol. It is expected that ${\beta _1}$ is negative. Similarly, ${\eta _1}$ can be interpreted as the total expenditure (income) elasticity, and it is expected that alcohol is a necessity such that $0<{\eta _1}<1$.

The double-log model naturally excludes any household that does not purchase alcohol. There are many reasons why we might observe non-drinking households in a cross-section survey dataset. The log specification precludes the use of Tobit estimation but, for robustness, a censored conditional quantile regression was also estimated, and yielded similar results. We further estimate the demand for on-premise and off-premise alcohol separately, with both prices featuring on the right-hand side, to test for substitution or complementarity between consumption at the two locations.

We also consider the quality of alcohol consumed. Our work on quality is based upon Deaton [24], which in turn builds on the seminal work by Prais and Houthakker [25]. Deaton’s work uses clusters to define prices, which for the purpose of this work will be regions $r$ at time $t$. True price variation is assumed not to occur within a cluster, such that all individuals in region $r$ at time $t$ face the same underlying price. However, they can select quality $q$. If we write expenditure as the product of price, quantity and quality, then ${X_{hrt}}={P_{rt}}{Q_{hrt}}{q_{hrt}}$. The theory begins with unit values, calculated by dividing expenditure by quantity. The unit value $V$ is then given as

2 $${V_{hrt}}=\frac{{{X_{hrt}}}}{{{Q_{hrt}}}}=\frac{{{P_{rt}}{Q_{hrt}}{q_{hrt}}}}{{{Q_{hrt}}}}={P_{rt}}{q_{hrt}}$$

which shows that the unit value is a combination of underlying price and an endogenously selected quality. As with Deaton, this can be rewritten as $\ln {V_{hrt}}=\ln {P_{rt}}+\ln {q_{hrt}}$. The price elasticity of quality demanded can be found by differentiating this with respect to $\ln {P_{rt}}$, which implies

$$\frac{{\partial \ln {V_{hrt}}}}{{\partial \ln {P_{rt}}}}=1+{\varepsilon _q}$$

where ${\varepsilon _q}$ is the price elasticity of quality demanded so that when price increases by 1%, the unit value increases by (1+${\varepsilon _q}$)%. To estimate the price elasticity of quality demanded, the regression equation is specified as

3 $$\ln {V_{hrt}}=\alpha +{\beta _2}\ln {P_{rt}}+{\eta _2}\ln {Y_{hrt}}+{\delta _2}\ln {A_{hrt}}+{\varvec{\gamma}_2}'{\varvec{Z}}+~{\theta _{hrt}}$$

where the same variables are used on the right-hand side as in the quantity demand equation. The price elasticity of quality demanded is calculated as $({\beta _2} - 1)$. It is expected that this price elasticity of quality demanded is negative, such that when prices increases consumers switch to lower quality alternatives, but that ${\beta _2}$ is itself positive. ${\eta _2}$ is expected to be positive since quality is expected to be a normal good. It is not a priori obvious whether it should be a luxury or a necessity.

Because we want to know how quality substitution occurs across the (quantity) distribution, the quality demand equation shown is estimated as a quantile regression, using the weights generated in the quantity quantile regression. Estimation using quantile regression on Eq. 3 would show how increase in price is passed on to unit values across the unit value distribution rather than the drinking distribution. The quality quantile regression is a quantile regression using weights, where the weights w at quantile τ are determined as

4 $${w_{hrt}}=\left\{ {\begin{array}{*{20}{l}} \tau &{{\text{if}}\quad {Q_{hrt}} \leqslant \widehat {{{Q_{hrt}}}}} \\ {1 - \tau }&{{\text{if}}\quad {Q_{hrt}}>\widehat {{{Q_{hrt}}}}} \end{array}} \right.$$

and $\widehat {{{Q_{hrt}}}}$ is the predicted value of the dependent variable ${Q_{hrt}}$ at quantile τ using quantile regression. Using the same weights from the quantity regression in the quality regression allows us to estimate the price elasticity of quality over the drinking distribution.

## Results

The tables presented in this section show, for brevity, only the price and income coefficients. Full results tables can be found in the “Appendix”. The results for the quantity decision are shown in Table 2—estimated by OLS (first column) and by quantile regression (in subsequent columns). These confirm our prior that price elasticity of demand at the top of the drinking distribution is less elastic than at the bottom of the distribution: the lower quartile’s price elasticity is − 0.709 compared to − 0.346 for the upper quartile. Unlike some findings in the literature, we find that no part of the drinking distribution is perfectly price inelastic. The income (total expenditure per capita) elasticity is positive, but less than 1, so that alcohol is a normal good. The effect of income is fairly constant across the drinking distribution. The positive coefficient on the log number of adults in the “Appendix” shows that for every extra adult in the household, household consumption increases but at a decreasing rate. The monthly fixed effects show that consumption increases significantly in November and December, whilst the North East and North West drink significantly more than any other region.

**Table 2:** Log price and income elasticities of log quantity demanded (a) all alcohol, (b) on-premise, (c) off-premise

	OLS	Q25	Q50	Q75	Q90	Q95
*(a)*						
Price	− 0.538*** (0.025)	− 0.709*** (0.053)	− 0.504*** (0.039)	− 0.346*** (0.034)	− 0.232*** (0.037)	− 0.176*** (0.043)
Income	0.344*** (0.014)	0.352*** (0.015)	0.388*** (0.011)	0.381*** (0.010)	0.340*** (0.010)	0.321*** (0.012)
Observations	54,069	54,069	54,069	54,069	54,069	54,069
*(b)*						
On-premise price	− 0.410*** (0.028)	− 0.455*** (0.049)	− 0.484*** (0.046)	− 0.361*** (0.047)	− 0.271*** (0.049)	− 0.273*** (0.059)
Off-premise price	0.040 (0.043)	0.081 (0.086)	0.044 (0.080)	− 0.021 (0.083)	− 0.073 (0.086)	− 0.067 (0.104)
Income	0.130*** (0.020)	0.149*** (0.016)	0.150*** (0.015)	0.128*** (0.015)	0.112*** (0.016)	0.140*** (0.019)
Observations	36,881	36,881	36,881	36,881	36,881	36,881
*(c)*						
On-premise price	0.041** (0.018)	0.060 (0.044)	0.050 (0.038)	− 0.029 (0.037)	− 0.000 (0.041)	0.036 (0.047)
Off-premise price	− 0.657*** (0.066)	− 0.881*** (0.079)	− 0.727*** (0.068)	− 0.460*** (0.066)	− 0.325*** (0.073)	− 0.222*** (0.083)
Income	0.311*** (0.013)	0.322*** (0.014)	0.340*** (0.012)	0.3442*** (0.012)	0.304*** (0.013)	0.286*** (0.014)
Observations	41,572	41,572	41,572	41,572	41,572	41,572

Significance: *p < 0.1, **p < 0.05, ***p < 0.01. Standard Errors in parentheses

**Fig. 3:**
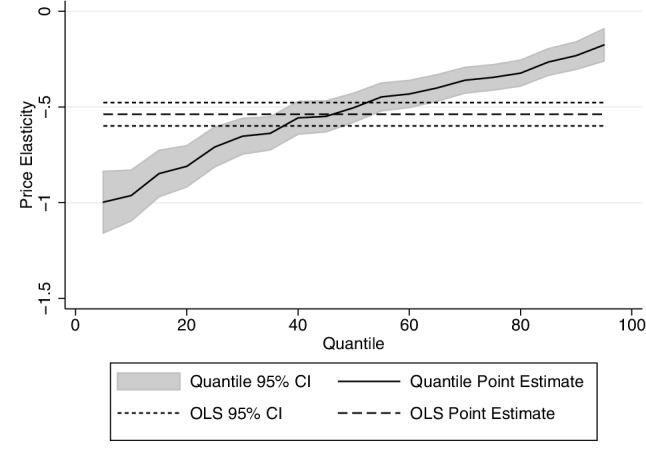
Price elasticity of quantity demand by qr: all alcohol

**Fig. 4:**
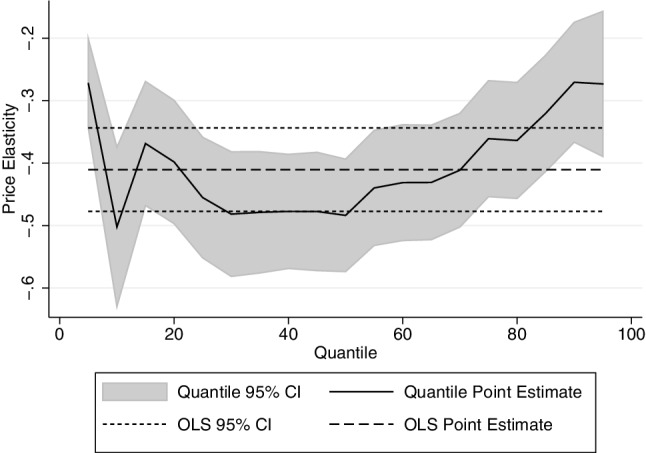
Price elasticity of quantity demand by qr: on-premise alcohol

**Fig. 5:**
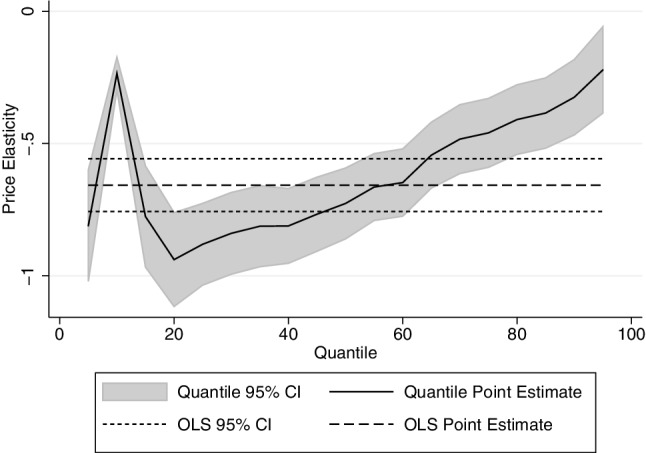
Price elasticity of quantity demand by qr: on-premise alcohol

The results for on- and off-premise alcohol are presented in Table 2b and c, respectively. They suggest that there is zero cross-price elasticity of demand. The demand for off-premise alcohol is more price elastic than the demand for on-premise alcohol. Perhaps surprisingly, the income elasticity is lower for on-premise alcohol. The detailed results in the “Appendix” show that seasonal effect is stronger in off-premise alcohol, with alcohol consumption increasing by 30% in December compared to January. As might perhaps be expected, households with more children consume less alcohol, especially on-trade alcohol (Figs. 3, 4, 5).

**Fig. 6:**
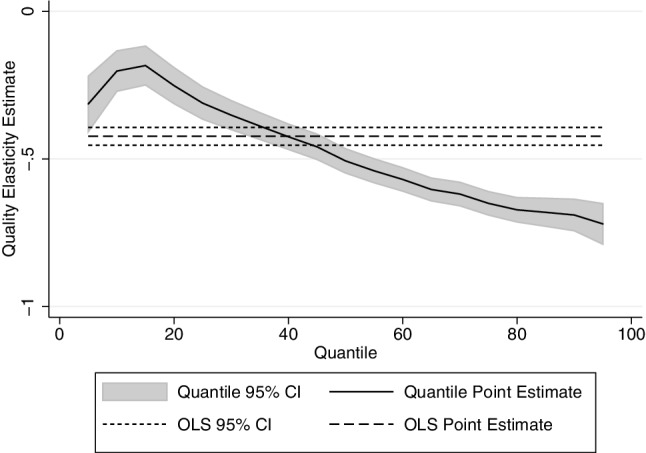
Price elasticity of quality demanded by qr: all alcohol

The results for the price and income elasticity of quality are shown in Table 3—again estimated by OLS (first column) and then by quantile regression. Note that these are estimated by quantiles of the quantity distribution—that is we use the same weights that are derived for the quantity equations. It is important to remember that the parameters shown below are the effect of price increases on price paid per unit, and as such the quality elasticities are calculated as ${\beta _2} - 1$. The results show that the price elasticity of quality demanded increase with consumption, such that heavier drinkers respond more to price changes by decreasing quality. The estimated price elasticity of demand (for all alcohol) by vingtile is shown in Fig. 6.

**Table 3:** Log price and income elasticities of log quality demanded (a) all alcohol, (b) on-premise, (c) off-premise

	OLS	Q25	Q50	Q75	Q90	Q95
*(a)*						
Price	0.577*** (0.015)	0.689*** (0.028)	0.493*** (0.021)	0.349*** (0.020)	0.310*** (0.027)	0.280*** (0.035)
Income	0.213*** (0.004)	0.206*** (0.007)	0.215*** (0.006)	0.215** (0.006)	0.234*** (0.008)	0.246*** (0.010)
Observations	54,017	54,017	54,017	54,017	54,017	54,017
*(b)*						
On-premise price	0.601*** (0.014)	0.427*** (0.015)	0.311*** (0.013)	0.233*** (0.013)	0.176*** (0.017)	0.176*** (0.017)
Off-premise price	− 0.021 (0.025)	0.009 (0.025)	0.027 (0.020)	0.050** (0.020)	0.030 (0.028)	0.023 (0.030)
Income	0.197*** (0.005)	0.158*** (0.005)	0.164*** (0.004)	0.166*** (0.004)	0.171*** (0.005)	0.179*** (0.005)
Observations	36,839	36,839	36,839	36,839	36,839	36,839
*(c)*						
On-premise price	− 0.022** (0.010)	− 0.018 (0.013)	− 0.024** (0.010)	− 0.028** (0.011)	− 0.021 (0.014)	− 0.014 (0.020)
Off-premise price	0.775*** (0.018)	0.730*** (0.023)	0.611*** (0.018)	0.541*** (0.021)	0.498*** (0.027)	0.461*** (0.036)
Income	0.134*** (0.003)	0.112*** (0.004)	0.124*** (0.003)	0.140*** (0.004)	0.150*** (0.005)	0.159*** (0.006)
Observations	41,487	41,487	41,487	41,487	41,487	41,487

Significance: *p < 0.1, **p < 0.05, ***p < 0.01. Standard Errors in parentheses

## Discussion

The results presented in this paper show convincingly that heavier drinkers are less responsive to price than moderate drinkers, especially for off-premise alcohol. The results seem plausible, and the OLS estimate for the price elasticity of demand for all alcohol lies within the range of the estimates found in the meta-analyses. The results also show that heavier drinkers respond to price increase by substituting with cheaper alcohol, which suggests that lighter drinkers are more brand loyal and do not choose their alcoholic beverages based on minimising price per unit paid.

These are important findings—the quantity results show that price-based measures will have little effect in reducing heavy consumption because of their small price elasticity, whilst simultaneously having a large negative effect on consumer surplus for the light drinking majority, because of their large price elasticity. Because heavy drinkers mitigate price increase by switching to lower quality, the price increase needed to reduce consumption is higher than might be expected when considering the mean elasticity.

The results of this paper have profound implications in the debate around minimum unit pricing. Modelling work on the effects of minimum pricing has used price elasticities where either heavy drinkers were more responsive [16] or at least as responsive [12] as moderate drinkers. The findings in this paper suggest that both studies are likely to have overestimated the health gains arising from minimum unit pricing. This problem is compounded by the increasing marginal rate of harm with consumption. The results from this study show that price increase is estimated to have a weaker effect on heavier drinkers than on moderate drinkers, so price-based alcohol policies may not be the most effective method of tackling heavy drinking without penalising moderate drinkers. That said, although the proportionate response is small for heavier drinkers (if the price goes up by 10% the upper quartile of reduce their consumption by 3.5%), the absolute number of units consumed decreases for the heaviest drinkers by more than the absolute decrease for lighter drinkers.

The price elasticity of quality demanded estimated in this paper shows that heavier drinkers respond to price increase by switching to lower quality alcohol. This can either be in the form of switching from on-premise alcohol consumption to off-premise alcohol consumption (where the unit value is lower), or by switching from one brand of drink to a cheaper alternative. While this makes little difference to health policy, unless of course lower quality alcohol is worse for health^5^, it has a major implication in the effect of price increases. If the heaviest drinkers absorb price increase by substituting towards lower quality alcohol, then price increase is less effective. Minimum unit pricing, which sets a floor price, may eliminate the possibility of absorbing price increase by switching to lower quality alcohol.

There are several limitations to this study. First, the fact that the data is collected at the household level means that some assumption must be made regarding the intra-household allocation of alcohol. Even though individual-level expenditure diaries are recorded, this is not sufficient to resolve this problem because of the possibility of intra-household transfers. This study thus implicitly assumes that the consumption of alcohol purchased within a household is split evenly.^6^ There may be cases where a household appears in the upper 5% of drinking households, while a very heavy drinker living in a large house with non-drinkers may not be included in the upper 5% of drinking households. This is perhaps unlikely to happen in a large amount of households because it relies on the other members of the household not drinking. The General Household Survey 2006 asks individuals how much alcohol they consume, and shows that in two-person households there is a strong positive correlation (0.30, p < 0.001) between individuals’ weekly units. This suggests that heavy drinking households are likely to be comprised of heavy drinking individuals rather than one heavy and one light drinker. Analysis at the individual level would be biased due to intra-household transfers, so would have its own limitations.

Second, stockpiling may mean that households purchase more at times when alcohol prices are lower, such as around December, but not consume it during the expenditure period. This is only relevant to off-premise alcohol consumption. Ideally, a longer diary window would reduce the problem of stockpiling, although this would likely cause lower response rates to the survey. Another option is to measure existing stocks at the start and end period to accurately measure consumption, as is done in a survey used by Gibson and Kim [26].

Third, under-recording of alcohol may bias the estimates. It is known that totals from household surveys reflect only around a half of alcohol clearances (see, for example, Boniface and Shelton [27]). However, it is not possible to know whether the under-recording is constant across the distribution, whether lighter drinkers under-record more than heavy drinkers, or the opposite. Under the first scenario, the estimates from this paper would be unbiased since constant under-recording in a double-log system simply appears as in the constant term. Similarly, measurement error in quantity may lead to bias because quantity is used to calculate price. However, no alternative measure of price is available in the survey.

## Conclusion

This paper has shown that heavier drinkers respond less to price in terms of quantity. If the price of alcohol increases by 10%, the lightest quartile of drinkers reduce their consumption by 7.1% compared to 3.5% for the heaviest quartile of drinkers. It is the first paper to examine quality substitution across the drinking distribution, and finds that heavier drinkers respond to price by changing quality more than lighter drinkers do.

The results suggest that price-based policies may not be effective at reducing consumption amongst heavy drinkers without penalising lighter drinkers. This is especially true if heavy drinkers can absorb price increase by decreasing quality. However, the presumed nonlinearity in consumption and harm means that a small proportional reduction in consumption amongst heavy drinkers could still have a large effect in terms of health and healthcare costs. The results also show that current modelling work on minimum unit pricing probably overstates the effects by not allowing the price elasticity to vary across the drinking distribution. This means that it predicts less consumer surplus loss for moderate drinkers, and a greater reduction in consumption in heavy drinkers, than would be expected given the results of this paper.

## Appendix

Full regression tables are found in Tables 4, 5, 6, 7, 8, 9. Table 10 shows the regression results using a monthly price index (with no regional variation).

**Table 4:** Log price and income effects on log quantity demanded: all alcohol

	(1)	(2)	(3)	(4)	(5)	(6)
	OLS	Q25	Q50	Q75	Q90	Q95
Log price per unit	− 0.538^***^ (0.025)	− 0.709^***^ (0.053)	− 0.504^***^ (0.039)	− 0.346^***^ (0.034)	− 0.232^***^ (0.037)	− 0.176^***^ (0.043)
Log per capita total expenditure	0.344^***^ (0.014)	0.352^***^ (0.015)	0.388^***^ (0.011)	0.381^***^ (0.010)	0.340^***^ (0.010)	0.321^***^ (0.012)
Log number of adults	0.714^***^ (0.022)	0.783^***^ (0.022)	0.762^***^ (0.016)	0.699^***^ (0.014)	0.625^***^ (0.015)	0.578^***^ (0.018)
Number of children	− 0.062^***^ (0.007)	− 0.077^***^ (0.010)	− 0.059^***^ (0.007)	− 0.057^***^ (0.006)	− 0.048^***^ (0.007)	− 0.044^***^ (0.008)
Age of oldest household member	− 0.001 (0.001)	− 0.003^***^ (0.001)	0.000 (0.000)	0.002^***^ (0.000)	0.004^***^ (0.000)	0.004^***^ (0.000)
February	0.096^***^ (0.017)	0.139^***^ (0.044)	0.111^***^ (0.032)	0.112^***^ (0.028)	0.111^***^ (0.030)	0.073^**^ (0.036)
March	0.099^**^ (0.032)	0.106^**^ (0.043)	0.109^***^ (0.032)	0.124^***^ (0.028)	0.102^***^ (0.030)	0.067^*^ (0.035)
April	0.133^***^ (0.019)	0.165^***^ (0.043)	0.148^***^ (0.031)	0.157^***^ (0.027)	0.107^***^ (0.029)	0.015 (0.035)
May	0.126^***^ (0.015)	0.155^***^ (0.043)	0.123^***^ (0.031)	0.134^***^ (0.027)	0.134^***^ (0.030)	0.095^***^ (0.035)
June	0.138^***^ (0.020)	0.199^***^ (0.043)	0.160^***^ (0.031)	0.142^***^ (0.027)	0.145^***^ (0.029)	0.099^***^ (0.035)
July	0.148^***^ (0.016)	0.195^***^ (0.042)	0.156^***^ (0.031)	0.127^***^ (0.027)	0.134^***^ (0.029)	0.106^***^ (0.034)
August	0.150^***^ (0.017)	0.183^***^ (0.042)	0.152^***^ (0.031)	0.142^***^ (0.027)	0.150^***^ (0.029)	0.067^*^ (0.034)
September	0.095^***^ (0.023)	0.129^***^ (0.042)	0.101^***^ (0.031)	0.104^***^ (0.027)	0.113^***^ (0.029)	0.040 (0.034)
October	0.111^***^ (0.027)	0.153^***^ (0.042)	0.137^***^ (0.031)	0.125^***^ (0.027)	0.119^***^ (0.029)	0.042 (0.034)
November	0.221^***^ (0.022)	0.255^***^ (0.042)	0.226^***^ (0.031)	0.214^***^ (0.027)	0.234^***^ (0.029)	0.198^***^ (0.034)
December	0.377^***^ (0.031)	0.450^***^ (0.043)	0.390^***^ (0.031)	0.356^***^ (0.027)	0.357^***^ (0.030)	0.310^***^ (0.035)
North West	− 0.019^***^ (0.001)	− 0.063 (0.048)	− 0.017 (0.035)	0.050^*^ (0.030)	0.016 (0.033)	0.015 (0.039)
Yorkshire and Humber	− 0.063^***^ (0.001)	− 0.114^**^ (0.049)	− 0.069^*^ (0.036)	− 0.010 (0.031)	− 0.011 (0.034)	− 0.022 (0.040)
East Midlands	− 0.126^***^ (0.002)	− 0.192^***^ (0.051)	− 0.147^***^ (0.037)	− 0.093^***^ (0.032)	− 0.049 (0.035)	− 0.033 (0.041)
West Midlands	− 0.141^***^ (0.002)	− 0.245^***^ (0.049)	− 0.131^***^ (0.036)	− 0.108^***^ (0.032)	− 0.051 (0.034)	− 0.042 (0.040)
Eastern	− 0.237^***^ (0.003)	− 0.320^***^ (0.050)	− 0.255^***^ (0.036)	− 0.190^***^ (0.032)	− 0.127^***^ (0.034)	− 0.100^**^ (0.040)
London	− 0.185^***^ (0.006)	− 0.253^***^ (0.053)	− 0.226^***^ (0.039)	− 0.177^***^	− 0.132^***^ (0.037)	− 0.117^***^ (0.043)
				(0.034)		
South East	− 0.199^***^ (0.004)	− 0.255^***^ (0.048)	− 0.232^***^ (0.035)	− 0.160^***^ (0.030)	− 0.122^***^ (0.033)	− 0.078^**^ (0.039)
South West	− 0.179^***^ (0.002)	− 0.246^***^ (0.049)	− 0.188^***^ (0.036)	− 0.121^***^ (0.031)	− 0.129^***^ (0.034)	− 0.110^***^ (0.040)
Wales	− 0.092^***^ (0.001)	− 0.119^**^ (0.055)	− 0.114^***^ (0.040)	− 0.064^*^ (0.035)	− 0.021 (0.038)	− 0.026 (0.045)
Scotland	− 0.057^***^ (0.003)	− 0.076 (0.050)	− 0.072^**^ (0.037)	− 0.032 (0.032)	− 0.011 (0.035)	− 0.028 (0.041)
Northern Ireland	− 0.024^***^ (0.007)	− 0.065 (0.055)	− 0.043 (0.040)	0.005 (0.035)	− 0.057 (0.038)	− 0.029 (0.045)
Linear monthly time trend	− 0.001^***^ (0.000)	− 0.001^***^ (0.000)	− 0.001^***^ (0.000)	− 0.001^***^ (0.000)	− 0.001^***^ (0.000)	− 0.000^***^ (0.000)
Constant	3.601^***^ (0.072)	3.614^***^ (0.216)	3.356^***^ (0.158)	3.367^***^ (0.138)	3.588^***^ (0.150)	3.769^***^ (0.176)
Observations	54,069	54,069	54,069	54,069	54,069	54,069

Significance: *0.1 **0.05 ***0.01. Standard errors in parentheses

**Table 5:** Log price and income effects on log quantity demanded: on-premise alcohol

	(1)	(2)	(3)	(4)	(5)	(6)
	OLS	Q25	Q50	Q75	Q90	Q95
Log price per unit on	− 0.410^***^ (0.028)	− 0.455^***^ (0.049)	− 0.484^***^ (0.046)	− 0.361^***^ (0.047)	− 0.271^***^ (0.049)	− 0.273^***^ (0.059)
Log price per unit off	0.040 (0.043)	0.081 (0.086)	0.044 (0.080)	− 0.021 (0.083)	− 0.073 (0.086)	− 0.067 (0.104)
Log per capita total expenditure	0.130^***^ (0.020)	0.149^***^ (0.016)	0.150^***^ (0.015)	0.128^***^ (0.015)	0.112^***^ (0.016)	0.140^***^ (0.019)
Log number of adults	0.691^***^ (0.017)	0.766^***^ (0.022)	0.823^***^ (0.021)	0.729^***^ (0.021)	0.552^***^ (0.022)	0.500^***^ (0.027)
Number of children	− 0.150^***^ (0.006)	− 0.158^***^ (0.010)	− 0.167^***^ (0.009)	− 0.159^***^ (0.009)	− 0.148^***^ (0.010)	− 0.142^***^ (0.012)
Age of oldest household member	− 0.010^***^ (0.001)	− 0.013^***^ (0.001)	− 0.012^***^ (0.001)	− 0.008^***^ (0.001)	− 0.006^***^ (0.001)	− 0.004^***^ (0.001)
February	0.040 (0.030)	0.046 (0.043)	0.022 (0.040)	0.075^*^ (0.042)	0.086^**^ (0.043)	0.028 (0.052)
March	0.038 (0.024)	0.034 (0.043)	0.021 (0.040)	0.053 (0.041)	0.109^**^ (0.043)	0.063 (0.052)
April	0.097^***^ (0.029)	0.128^***^ (0.042)	0.120^***^ (0.039)	0.087^**^ (0.040)	0.089^**^ (0.042)	0.091^*^ (0.051)
May	0.061^**^ (0.026)	0.064 (0.042)	0.059 (0.040)	0.082^**^ (0.041)	0.046 (0.042)	0.048 (0.051)
June	0.036 (0.043)	− 0.002 (0.042)	0.027 (0.039)	0.092^**^ (0.040)	0.069^*^ (0.042)	0.030 (0.051)
July	0.072^**^ (0.027)	0.039 (0.042)	0.092^**^ (0.039)	0.096^**^ (0.040)	0.070^*^ (0.042)	0.045 (0.050)
August	0.095^***^ (0.024)	0.084^**^ (0.042)	0.102^***^ (0.039)	0.123^***^ (0.040)	0.099^**^ (0.042)	0.040 (0.050)
September	0.039 (0.025)	0.058 (0.042)	0.027 (0.039)	0.052 (0.040)	0.034 (0.042)	0.006 (0.051)
October	0.024 (0.034)	− 0.005 (0.042)	0.023 (0.039)	0.072^*^ (0.041)	0.090^**^ (0.042)	0.051 (0.051)
November	0.026 (0.024)	0.033 (0.042)	0.005 (0.039)	0.049 (0.040)	0.068 (0.042)	0.046 (0.051)
December	0.107^***^ (0.033)	0.120^***^ (0.043)	0.111^***^ (0.040)	0.101^**^ (0.042)	0.147^***^ (0.043)	0.110^**^ (0.052)
Northwest	− 0.100^***^ (0.004)	− 0.094^**^ (0.047)	− 0.159^***^ (0.044)	− 0.115^**^ (0.045)	− 0.022 (0.047)	0.049 (0.057)
Yorkshire and Humber	− 0.108^***^ (0.004)	− 0.110^**^ (0.049)	− 0.152^***^ (0.045)	− 0.139^***^ (0.047)	− 0.005 (0.048)	0.021 (0.058)
East Midlands	− 0.246^***^ (0.004)	− 0.273^***^ (0.050)	− 0.299^***^ (0.047)	− 0.274^***^ (0.048)	− 0.162^***^ (0.050)	− 0.116^*^ (0.060)
West Midlands	− 0.190^***^ (0.004)	− 0.173^***^ (0.049)	− 0.234^***^ (0.046)	− 0.222^***^ (0.047)	− 0.122^**^ (0.049)	− 0.099^*^ (0.059)
Eastern	− 0.368^***^ (0.009)	− 0.349^***^ (0.050)	− 0.433^***^ (0.047)	− 0.431^***^ (0.049)	− 0.319^***^ (0.050)	− 0.223^***^ (0.061)
London	− 0.208^***^ (0.015)	− 0.211^***^ (0.056)	− 0.255^***^ (0.052)	− 0.234^***^ (0.054)	− 0.112^**^ (0.056)	− 0.090 (0.068)
Southeast	− 0.352^***^ (0.011)	− 0.319^***^ (0.049)	− 0.431^***^ (0.046)	− 0.444^***^ (0.047)	− 0.288^***^ (0.049)	− 0.220^***^ (0.059)
Southwest	− 0.312^***^ (0.007)	− 0.294^***^ (0.049)	− 0.365^***^ (0.046)	− 0.372^***^ (0.047)	− 0.243^***^ (0.049)	− 0.234^***^ (0.059)
Wales	− 0.138^***^ (0.003)	− 0.172^***^ (0.055)	− 0.181^***^ (0.051)	− 0.152^***^ (0.053)	− 0.128^**^ (0.055)	− 0.099 (0.066)
Scotland	− 0.220^***^ (0.009)	− 0.185^***^ (0.052)	− 0.254^***^ (0.048)	− 0.282^***^ (0.050)	− 0.194^***^ (0.051)	− 0.191^***^ (0.062)
Northern Ireland	− 0.104^***^ (0.012)	− 0.141^**^ (0.056)	− 0.128^**^ (0.053)	− 0.136^**^ (0.054)	− 0.020 (0.056)	0.019 (0.068)
Linear monthly time trend	− 0.002^***^ (0.000)	− 0.001^***^ (0.000)	− 0.002^***^ (0.000)	− 0.003^***^ (0.000)	− 0.003^***^ (0.000)	− 0.003^***^ (0.000)
Constant	4.051^***^ (0.180)	3.269^***^ (0.333)	4.405^***^ (0.311)	4.854^***^ (0.321)	5.211^***^ (0.331)	5.304^***^ (0.401)
Observations	36,881	36,881	36,881	36,881	36,881	36,881

Significance: *0.1 **0.05 ***0.01. Standard errors in parentheses

**Table 6:** Log price and income effects on log quantity demanded: off-premise alcohol

	(1)	(2)	(3)	(4)	(5)	(6)
	OLS	Q25	Q50	Q75	Q90	Q95
Log price per unit on	0.041^**^ (0.018)	0.060 (0.044)	0.050 (0.038)	− 0.029 (0.037)	− 0.000 (0.041)	0.036 (0.047)
Log price per unit off	− 0.657^***^ (0.066)	− 0.881^***^ (0.079)	− 0.727^***^ (0.068)	− 0.460^***^ (0.066)	− 0.325^***^ (0.073)	− 0.222^***^ (0.083)
Log per capita total expenditure	0.311^***^ (0.013)	0.322^***^ (0.014)	0.340^***^ (0.012)	0.342^***^ (0.012)	0.304^***^ (0.013)	0.286^***^ (0.014)
Log number of adults	0.468^***^ (0.019)	0.459^***^ (0.020)	0.519^***^ (0.018)	0.552^***^ (0.017)	0.525^***^ (0.019)	0.508^***^ (0.021)
Number of children	− 0.016^*^ (0.008)	− 0.018^**^ (0.009)	− 0.016^**^ (0.008)	− 0.013^*^ (0.007)	− 0.015^*^ (0.008)	− 0.019^**^ (0.009)
Age of oldest household member	0.005^***^ (0.001)	0.005^***^ (0.001)	0.006^***^ (0.000)	0.007^***^ (0.000)	0.008^***^ (0.001)	0.007^***^ (0.001)
February	0.111^***^ (0.030)	0.164^***^ (0.041)	0.116^***^ (0.036)	0.117^***^ (0.035)	0.120^***^ (0.038)	0.050 (0.043)
March	0.086^**^ (0.030)	0.118^***^ (0.040)	0.100^***^ (0.035)	0.082^**^ (0.034)	0.085^**^ (0.037)	0.003 (0.043)
April	0.082^***^ (0.023)	0.130^***^ (0.040)	0.096^***^ (0.034)	0.106^***^ (0.033)	0.076^**^ (0.036)	− 0.044 (0.042)
May	0.090^***^ (0.018)	0.140^***^ (0.040)	0.093^***^ (0.034)	0.096^***^ (0.033)	0.135^***^ (0.036)	0.077^*^ (0.042)
June	0.119^***^ (0.020)	0.182^***^ (0.039)	0.120^***^ (0.034)	0.110^***^ (0.033)	0.108^***^ (0.036)	0.052 (0.041)
July	0.103^***^ (0.025)	0.119^***^ (0.039)	0.115^***^ (0.034)	0.098^***^ (0.033)	0.109^***^ (0.036)	0.039 (0.041)
August	0.096^***^ (0.025)	0.122^***^ (0.039)	0.098^***^ (0.034)	0.092^***^ (0.033)	0.116^***^ (0.036)	0.014 (0.041)
September	0.063^***^ (0.017)	0.068^*^ (0.039)	0.089^***^ (0.034)	0.074^**^ (0.033)	0.088^**^ (0.036)	− 0.007 (0.042)
October	0.102^***^ (0.028)	0.119^***^ (0.039)	0.116^***^ (0.034)	0.096^***^ (0.033)	0.105^***^ (0.036)	− 0.006 (0.041)
November	0.221^***^ (0.024)	0.257^***^ (0.038)	0.226^***^ (0.033)	0.209^***^ (0.032)	0.256^***^ (0.035)	0.188^***^ (0.040)
December	0.376^***^ (0.029)	0.442^***^ (0.039)	0.396^***^ (0.033)	0.376^***^ (0.032)	0.389^***^ (0.035)	0.295^***^ (0.041)
Northwest	0.037^***^ (0.005)	0.040 (0.044)	0.048 (0.038)	0.056 (0.037)	0.030 (0.040)	− 0.008 (0.046)
Yorkshire and Humber	− 0.016^***^ (0.005)	− 0.020 (0.046)	− 0.020 (0.039)	− 0.017 (0.038)	− 0.028 (0.042)	− 0.030 (0.048)
East Midlands	− 0.055^***^ (0.005)	− 0.094^**^ (0.047)	− 0.078^*^ (0.040)	− 0.067^*^ (0.039)	− 0.022 (0.043)	0.000 (0.049)
West Midlands	− 0.068^***^ (0.005)	− 0.088^*^ (0.046)	− 0.053 (0.039)	− 0.044 (0.038)	− 0.011 (0.042)	− 0.031 (0.048)
Eastern	− 0.104^***^ (0.010)	− 0.131^***^ (0.047)	− 0.111^***^ (0.040)	− 0.097^**^ (0.039)	− 0.074^*^ (0.043)	− 0.079 (0.049)
London	− 0.136^***^ (0.016)	− 0.166^***^ (0.052)	− 0.169^***^ (0.045)	− 0.136^***^ (0.044)	− 0.115^**^ (0.048)	− 0.130^**^ (0.055)
Southeast	− 0.075^***^ (0.012)	− 0.081^*^ (0.045)	− 0.090^**^ (0.039)	− 0.085^**^ (0.038)	− 0.052 (0.042)	− 0.052 (0.048)
Southwest	− 0.056^***^ (0.008)	− 0.055 (0.045)	− 0.072^*^ (0.039)	− 0.057 (0.038)	− 0.077^*^ (0.042)	− 0.094^*^ (0.048)
Wales	− 0.030^***^ (0.004)	− 0.048 (0.051)	− 0.016 (0.044)	− 0.020 (0.043)	0.002 (0.047)	− 0.026 (0.054)
Scotland	0.037^***^ (0.008)	0.042 (0.047)	0.046 (0.041)	0.039 (0.040)	0.048 (0.044)	0.012 (0.050)
Northern Ireland	0.038^***^ (0.011)	0.069 (0.053)	0.045 (0.046)	0.056 (0.044)	− 0.012 (0.049)	− 0.042 (0.056)
Linear monthly time trend	− 0.000^***^ (0.000)	− 0.001^***^ (0.000)	− 0.000^***^ (0.000)	− 0.000^**^ (0.000)	− 0.000^*^ (0.000)	0.000 (0.000)
Constant	3.363^***^ (0.230)	3.238^***^ (0.304)	3.411^***^ (0.263)	3.501^***^ (0.256)	3.625^***^ (0.280)	3.614^***^ (0.321)
Observations	41,572	41,572	41,572	41,572	41,572	41,572

Significance: *0.1 **0.05 ***0.01. Standard errors in parentheses

**Table 7:** Log price and income effects on log quality demanded: all alcohol

	(1)	(2)	(3)	(4)	(5)	(6)
	OLS	Q25	Q50	Q75	Q90	Q95
Log price per unit	0.577^***^ (0.015)	0.689^***^ (0.028)	0.493^***^ (0.021)	0.349^***^ (0.020)	0.310^***^ (0.027)	0.280^***^ (0.035)
Log per capita total expenditure	0.213^***^ (0.004)	0.206^***^ (0.007)	0.215^***^ (0.006)	0.215^***^ (0.006)	0.234^***^ (0.008)	0.246^***^ (0.010)
Log number of adults	0.139^***^ (0.006)	0.134^***^ (0.011)	0.171^***^ (0.008)	0.164^***^ (0.008)	0.144^***^ (0.011)	0.148^***^ (0.015)
Number of children	− 0.094^***^ (0.003)	− 0.109^***^ (0.005)	− 0.111^***^ (0.004)	− 0.108^***^ (0.003)	− 0.101^***^ (0.004)	− 0.090^***^ (0.006)
Age of oldest household member	− 0.005^***^ (0.000)	− 0.005^***^ (0.000)	− 0.006^***^ (0.000)	− 0.006^***^ (0.000)	− 0.006^***^ (0.000)	− 0.005^***^ (0.000)
February	− 0.016 (0.013)	− 0.017 (0.024)	− 0.022 (0.018)	− 0.018 (0.020)	− 0.001 (0.022)	0.009 (0.028)
March	− 0.011 (0.013)	− 0.020 (0.023)	− 0.027 (0.017)	− 0.018 (0.018)	0.003 (0.023)	− 0.016 (0.029)
April	− 0.020 (0.012)	− 0.058^**^ (0.023)	− 0.029^*^ (0.017)	− 0.024 (0.018)	− 0.015 (0.023)	0.027 (0.031)
May	− 0.016 (0.013)	− 0.022 (0.025)	− 0.039^**^ (0.017)	− 0.032^*^ (0.018)	− 0.002 (0.023)	− 0.001 (0.030)
June	− 0.028^**^ (0.012)	− 0.053^**^ (0.024)	− 0.046^***^ (0.016)	− 0.022 (0.018)	− 0.006 (0.023)	0.000 (0.029)
July	− 0.026^**^ (0.012)	− 0.057^**^ (0.023)	− 0.045^***^ (0.017)	− 0.049^***^ (0.018)	− 0.021 (0.021)	0.000 (0.028)
August	− 0.026^**^ (0.012)	− 0.056^**^ (0.023)	− 0.043^**^ (0.017)	− 0.035^*^ (0.018)	− 0.027 (0.022)	− 0.001 (0.028)
September	− 0.018 (0.012)	− 0.036 (0.023)	− 0.032^*^ (0.017)	− 0.031^*^ (0.019)	0.002 (0.025)	0.031 (0.033)
October	− 0.034^***^ (0.012)	− 0.071^***^ (0.023)	− 0.055^***^ (0.017)	− 0.040^**^ (0.019)	− 0.011 (0.022)	0.025 (0.032)
November	− 0.049^***^ (0.012)	− 0.087^***^ (0.024)	− 0.083^***^ (0.016)	− 0.071^***^ (0.018)	− 0.049^**^ (0.024)	− 0.048 (0.031)
December	− 0.073^***^ (0.013)	− 0.127^***^ (0.023)	− 0.119^***^ (0.016)	− 0.105^***^ (0.018)	− 0.084^***^ (0.023)	− 0.086^***^ (0.029)
Northwest	− 0.017 (0.014)	− 0.029 (0.022)	− 0.040^***^ (0.015)	− 0.021 (0.016)	− 0.004 (0.023)	0.009 (0.031)
Yorkshire and Humber	0.001 (0.014)	0.010 (0.021)	0.004 (0.016)	0.020 (0.016)	0.043^*^ (0.025)	0.036 (0.032)
East Midlands	− 0.012 (0.015)	− 0.008 (0.023)	− 0.018 (0.016)	− 0.009 (0.017)	0.014 (0.024)	0.008 (0.031)
West Midlands	− 0.001 (0.014)	0.022 (0.022)	0.004 (0.017)	0.003 (0.017)	0.009 (0.023)	0.028 (0.032)
Eastern	− 0.023 (0.014)	− 0.024 (0.023)	− 0.032^*^ (0.016)	− 0.036^**^ (0.016)	− 0.024 (0.023)	− 0.035 (0.032)
London	− 0.002 (0.015)	− 0.027 (0.025)	0.006 (0.017)	0.057^***^ (0.019)	0.098^***^ (0.025)	0.109^***^ (0.034)
Southeast	− 0.035^**^ (0.014)	− 0.045^**^ (0.022)	− 0.055^***^ (0.016)	− 0.031^*^ (0.016)	− 0.009 (0.023)	0.003 (0.031)
Southwest	− 0.012 (0.014)	− 0.009 (0.023)	− 0.029^*^ (0.016)	− 0.014 (0.015)	0.010 (0.024)	0.006 (0.030)
Wales	0.007 (0.016)	− 0.012 (0.027)	− 0.019 (0.018)	− 0.009 (0.016)	0.021 (0.026)	0.044 (0.035)
Scotland	− 0.021 (0.015)	− 0.051^**^ (0.025)	− 0.056^***^ (0.016)	− 0.046^***^ (0.017)	− 0.035 (0.024)	− 0.019 (0.029)
Northern Ireland	0.019 (0.016)	− 0.030 (0.030)	− 0.013 (0.019)	0.003 (0.021)	0.026 (0.027)	0.057 (0.035)
Linear monthly time trend	− 0.000^**^ (0.000)	− 0.000^***^ (0.000)	− 0.001^***^ (0.000)	− 0.001^***^ (0.000)	− 0.001^***^ (0.000)	− 0.001^***^ (0.000)
Constant	0.642^***^ (0.063)	0.375^***^ (0.112)	0.984^***^ (0.084)	1.430^***^ (0.082)	1.363^***^ (0.111)	1.314^***^ (0.143)
Observations	54,017	54,017	54,017	54,017	54,017	54,017

Significance: *0.1 **0.05 ***0.01. Standard errors in parentheses

**Table 8:** Log price and income effects on log quality demanded: on-premise alcohol

	(1)	(2)	(3)	(4)	(5)	(6)
	OLS	Q25	Q50	Q75	Q90	Q95
Log price per unit on	0.601^***^ (0.014)	0.427^***^ (0.015)	0.311^***^ (0.013)	0.233^***^ (0.013)	0.176^***^ (0.017)	0.176^***^ (0.017)
Log price per unit off	− 0.021 (0.025)	0.009 (0.025)	0.027 (0.020)	0.050^**^ (0.020)	0.030 (0.028)	0.023 (0.030)
Log per capita total expenditure	0.197^***^ (0.005)	0.158^***^ (0.005)	0.164^***^ (0.004)	0.166^***^ (0.004)	0.171^***^ (0.005)	0.179^***^ (0.005)
Log number of adults	0.051^***^ (0.006)	0.047^***^ (0.006)	0.060^***^ (0.005)	0.076^***^ (0.005)	0.079^***^ (0.007)	0.073^***^ (0.007)
Number of children	− 0.032^***^ (0.003)	− 0.029^***^ (0.003)	− 0.028^***^ (0.002)	− 0.030^***^ (0.002)	− 0.026^***^ (0.003)	− 0.022^***^ (0.004)
Age of oldest household member	− 0.002^***^ (0.000)	− 0.002^***^ (0.000)	− 0.002^***^ (0.000)	− 0.002^***^ (0.000)	− 0.002^***^ (0.000)	− 0.002^***^ (0.000)
February	− 0.013 (0.012)	0.005 (0.012)	− 0.009 (0.010)	− 0.012 (0.009)	− 0.030^**^ (0.013)	− 0.055^***^ (0.017)
March	− 0.012 (0.012)	0.011 (0.012)	0.004 (0.009)	− 0.003 (0.010)	− 0.030^**^ (0.014)	− 0.028^*^ (0.015)
April	− 0.010 (0.012)	− 0.007 (0.011)	0.001 (0.009)	− 0.007 (0.009)	− 0.030^**^ (0.014)	− 0.025^*^ (0.015)
May	− 0.010 (0.012)	− 0.007 (0.011)	− 0.008 (0.009)	− 0.020^**^ (0.009)	− 0.033^**^ (0.013)	− 0.027^**^ (0.013)
June	− 0.022^*^ (0.012)	− 0.016 (0.011)	− 0.018^**^ (0.009)	− 0.020^**^ (0.009)	− 0.025^**^ (0.013)	− 0.031^**^ (0.013)
July	− 0.020^*^ (0.012)	− 0.017 (0.011)	− 0.014 (0.010)	− 0.016^*^ (0.009)	− 0.033^**^ (0.014)	− 0.043^***^ (0.014)
August	− 0.021^*^ (0.012)	0.003 (0.011)	− 0.007 (0.009)	− 0.018^*^ (0.009)	− 0.029^**^ (0.013)	− 0.029^**^ (0.014)
September	− 0.010 (0.012)	− 0.003 (0.010)	− 0.005 (0.009)	− 0.010 (0.010)	− 0.017 (0.012)	− 0.014 (0.013)
October	− 0.015 (0.012)	− 0.010 (0.011)	− 0.021^**^ (0.009)	− 0.024^***^ (0.009)	− 0.037^***^ (0.013)	− 0.037^**^ (0.015)
November	− 0.013 (0.012)	0.008 (0.013)	− 0.007 (0.010)	− 0.020^**^ (0.009)	− 0.033^***^ (0.013)	− 0.031^*^ (0.016)
December	− 0.014 (0.012)	0.012 (0.013)	0.009 (0.010)	− 0.007 (0.010)	− 0.019 (0.013)	− 0.038^***^ (0.013)
Northwest	0.002 (0.014)	0.005 (0.012)	0.023^**^ (0.010)	0.035^***^ (0.012)	0.033^*^ (0.018)	0.035^**^ (0.018)
Yorkshire and Humber	− 0.004 (0.014)	− 0.005 (0.012)	0.009 (0.010)	0.020^*^ (0.012)	0.027 (0.018)	0.037^**^ (0.017)
East Midlands	0.020 (0.014)	0.012 (0.013)	0.037^***^ (0.011)	0.057^***^ (0.012)	0.070^***^ (0.017)	0.094^***^ (0.015)
West Midlands	0.020 (0.014)	0.019 (0.011)	0.039^***^ (0.011)	0.053^***^ (0.012)	0.053^***^ (0.017)	0.068^***^ (0.016)
Eastern	0.028^*^ (0.015)	0.043^***^ (0.014)	0.081^***^ (0.012)	0.113^***^ (0.013)	0.133^***^ (0.018)	0.150^***^ (0.017)
London	0.040^**^ (0.016)	0.099^***^ (0.018)	0.135^***^ (0.014)	0.160^***^ (0.016)	0.165^***^ (0.022)	0.180^***^ (0.021)
Southeast	0.027^*^ (0.014)	0.055^***^ (0.013)	0.102^***^ (0.011)	0.136^***^ (0.013)	0.145^***^ (0.019)	0.154^***^ (0.018)
Southwest	0.031^**^ (0.014)	0.038^***^ (0.013)	0.077^***^ (0.011)	0.102^***^ (0.012)	0.115^***^ (0.018)	0.135^***^ (0.017)
Wales	0.028^*^ (0.016)	0.034^**^ (0.015)	0.046^***^ (0.012)	0.059^***^ (0.013)	0.073^***^ (0.020)	0.071^***^ (0.015)
Scotland	0.048^***^ (0.015)	0.073^***^ (0.017)	0.103^***^ (0.013)	0.121^***^ (0.015)	0.118^***^ (0.019)	0.117^***^ (0.021)
Northern Ireland	0.079^***^ (0.016)	0.141^***^ (0.016)	0.161^***^ (0.016)	0.152^***^ (0.016)	0.140^***^ (0.023)	0.132^***^ (0.021)
Linear monthly time trend	0.000^***^ (0.000)	0.001^***^ (0.000)	0.001^***^ (0.000)	0.001^***^ (0.000)	0.001^***^ (0.000)	0.001^***^ (0.000)
Constant	0.745^***^ (0.096)	1.516^***^ (0.091)	1.864^***^ (0.080)	2.066^***^ (0.079)	2.305^***^ (0.106)	2.265^***^ (0.114)
Observations	36,839	36,839	36,839	36,839	36,839	36,839

Significance: *0.1 **0.05 ***0.01. Standard errors in parentheses

**Table 9:** Log price and income effects on log quality demanded: off-premise

	(1)	(2)	(3)	(4)	(5)	(6)
	OLS	Q25	Q50	Q75	Q90	Q95
Log price per unit on	− 0.022^**^ (0.010)	− 0.018 (0.013)	− 0.024^**^ (0.010)	− 0.028^**^ (0.011)	− 0.021 (0.014)	− 0.014 (0.020)
Log price per unit off	0.775^***^ (0.018)	0.730^***^ (0.023)	0.611^***^ (0.018)	0.541^***^ (0.021)	0.498^***^ (0.027)	0.461^***^ (0.036)
Log per capita total expenditure	0.134^***^ (0.003)	0.112^***^ (0.004)	0.124^***^ (0.003)	0.140^***^ (0.004)	0.150^***^ (0.005)	0.159^***^ (0.006)
Log number of adults	0.033^***^ (0.005)	0.012^**^ (0.006)	0.024^***^ (0.005)	0.031^***^ (0.005)	0.036^***^ (0.007)	0.034^***^ (0.009)
Number of children	− 0.024^***^ (0.002)	− 0.025^***^ (0.003)	− 0.024^***^ (0.002)	− 0.022^***^ (0.002)	− 0.020^***^ (0.003)	− 0.018^***^ (0.004)
Age of oldest household member	− 0.001^***^ (0.000)	− 0.002^***^ (0.000)	− 0.002^***^ (0.000)	− 0.001^***^ (0.000)	− 0.001^***^ (0.000)	− 0.001^***^ (0.000)
February	− 0.004 (0.009)	− 0.005 (0.012)	− 0.008 (0.010)	− 0.016 (0.010)	− 0.010 (0.015)	− 0.015 (0.019)
March	0.003 (0.009)	− 0.004 (0.012)	0.001 (0.008)	− 0.010 (0.010)	− 0.005 (0.013)	− 0.020 (0.016)
April	− 0.004 (0.009)	− 0.013 (0.012)	− 0.005 (0.008)	− 0.013 (0.010)	− 0.012 (0.014)	− 0.031^*^ (0.016)
May	0.001 (0.009)	0.008 (0.012)	− 0.003 (0.009)	− 0.010 (0.010)	− 0.018 (0.013)	− 0.021 (0.018)
June	− 0.003 (0.009)	0.003 (0.012)	0.001 (0.009)	− 0.002 (0.010)	− 0.020 (0.015)	− 0.026 (0.017)
July	− 0.005 (0.009)	− 0.013 (0.012)	− 0.006 (0.009)	− 0.015 (0.011)	− 0.009 (0.013)	− 0.020 (0.016)
August	− 0.006 (0.009)	− 0.002 (0.012)	− 0.008 (0.009)	− 0.017^*^ (0.010)	− 0.011 (0.012)	− 0.024 (0.016)
September	0.000 (0.009)	− 0.011 (0.012)	− 0.005 (0.008)	− 0.006 (0.010)	− 0.001 (0.014)	− 0.012 (0.019)
October	0.001 (0.009)	− 0.003 (0.012)	− 0.001 (0.008)	− 0.010 (0.010)	− 0.017 (0.015)	− 0.031^**^ (0.015)
November	− 0.008 (0.009)	− 0.014 (0.012)	− 0.018^**^ (0.008)	− 0.018^*^ (0.010)	− 0.013 (0.013)	− 0.014 (0.018)
December	− 0.017^*^ (0.009)	− 0.018 (0.013)	− 0.018^**^ (0.008)	− 0.013 (0.010)	− 0.010 (0.013)	− 0.037^**^ (0.017)
Northwest	− 0.007 (0.010)	− 0.008 (0.011)	− 0.004 (0.009)	0.000 (0.009)	0.011 (0.013)	− 0.015 (0.019)
Yorkshire and Humber	− 0.004 (0.010)	− 0.006 (0.012)	0.000 (0.009)	0.004 (0.010)	0.012 (0.013)	− 0.008 (0.020)
East Midlands	− 0.007 (0.010)	0.003 (0.012)	0.007 (0.009)	0.015 (0.009)	0.023 (0.014)	0.005 (0.020)
West Midlands	− 0.001 (0.010)	0.003 (0.012)	0.003 (0.010)	0.002 (0.010)	− 0.003 (0.015)	− 0.018 (0.018)
Eastern	− 0.009 (0.010)	− 0.001 (0.011)	0.007 (0.010)	0.011 (0.010)	0.023 (0.014)	0.012 (0.022)
London	− 0.005 (0.012)	0.002 (0.014)	0.032^***^ (0.011)	0.051^***^ (0.012)	0.063^***^ (0.018)	0.059^**^ (0.024)
Southeast	− 0.016 (0.010)	− 0.009 (0.012)	0.006 (0.010)	0.013 (0.010)	0.022 (0.015)	− 0.005 (0.020)
Southwest	− 0.005 (0.010)	− 0.007 (0.011)	0.002 (0.009)	0.012 (0.010)	0.024^*^ (0.013)	0.006 (0.019)
Wales	0.003 (0.011)	− 0.002 (0.013)	0.001 (0.011)	0.007 (0.012)	0.013 (0.017)	− 0.011 (0.021)
Scotland	0.008 (0.011)	0.001 (0.012)	0.013 (0.010)	0.018^*^ (0.011)	0.026^*^ (0.014)	− 0.006 (0.020)
Northern Ireland	0.020^*^ (0.012)	0.040^***^ (0.015)	0.046^***^ (0.011)	0.045^***^ (0.011)	0.058^***^ (0.016)	0.044^*^ (0.023)
Linear monthly time trend	0.000^***^ (0.000)	0.000^*^	0.000^**^	0.000	− 0.000	0.000
		(0.000)	(0.000)	(0.000)	(0.000)	(0.000)
Constant	0.155^**^ (0.068)	0.453^***^ (0.089)	0.724^***^ (0.070)	0.823^***^ (0.077)	0.822^***^ (0.104)	0.857^***^ (0.138)
Observations	41,487	41,487	41,487	41,487	41,487	41,487

Significance: *0.1 **0.05 ***0.01. Standard errors in parentheses

**Table 10:** Log price and income effects on log quantity demanded: all alcohol (robustness check, monthly price)

	(1)	(2)	(3)	(4)	(5)	(6)
	OLS	Q25	Q50	Q75	Q90	Q95
Log price per unit	− 0.570^***^ (0.071)	− 0.762^***^ (0.155)	− 0.560^***^ (0.116)	− 0.423^***^ (0.101)	− 0.314^***^ (0.108)	− 0.272^**^ (0.130)
Log per capita total expenditure	0.338^***^ (0.014)	0.349^***^ (0.015)	0.385^***^ (0.011)	0.380^***^ (0.010)	0.336^***^ (0.010)	0.322^***^ (0.013)
Log number of adults	0.712^***^ (0.023)	0.777^***^ (0.022)	0.759^***^ (0.016)	0.704^***^ (0.014)	0.625^***^ (0.015)	0.578^***^ (0.018)
Number of children	− 0.060^***^ (0.007)	− 0.075^***^ (0.010)	− 0.060^***^ (0.007)	− 0.058^***^ (0.006)	− 0.048^***^ (0.007)	− 0.045^***^ (0.008)
Age of oldest household member	− 0.000 (0.001)	− 0.003^***^ (0.001)	0.001 (0.000)	0.002^***^ (0.000)	0.004^***^ (0.000)	0.004^***^ (0.001)
February	0.098^***^ (0.018)	0.125^***^ (0.043)	0.110^***^ (0.032)	0.117^***^ (0.028)	0.118^***^ (0.030)	0.097^***^ (0.036)
March	0.101^***^ (0.031)	0.106^**^ (0.043)	0.124^***^ (0.032)	0.121^***^ (0.028)	0.107^***^ (0.030)	0.073^**^ (0.036)
April	0.137^***^ (0.018)	0.159^***^ (0.042)	0.165^***^ (0.031)	0.155^***^ (0.028)	0.106^***^ (0.029)	0.027 (0.035)
May	0.122^***^ (0.021)	0.140^***^ (0.043)	0.126^***^ (0.032)	0.123^***^ (0.028)	0.126^***^ (0.030)	0.107^***^ (0.036)
June	0.139^***^ (0.032)	0.190^***^ (0.043)	0.158^***^ (0.032)	0.135^***^ (0.028)	0.142^***^ (0.030)	0.104^***^ (0.036)
July	0.148^***^ (0.018)	0.190^***^ (0.042)	0.154^***^ (0.032)	0.123^***^ (0.028)	0.127^***^ (0.029)	0.115^***^ (0.035)
August	0.151^***^ (0.017)	0.176^***^ (0.042)	0.152^***^ (0.032)	0.135^***^ (0.028)	0.145^***^ (0.030)	0.079^**^ (0.035)
September	0.098^**^ (0.033)	0.133^***^ (0.042)	0.102^***^ (0.031)	0.098^***^ (0.027)	0.109^***^ (0.029)	0.044 (0.035)
October	0.112^***^ (0.029)	0.148^***^ (0.043)	0.137^***^ (0.032)	0.118^***^ (0.028)	0.124^***^ (0.030)	0.051 (0.036)
November	0.219^***^ (0.026)	0.251^***^ (0.044)	0.230^***^ (0.033)	0.211^***^ (0.029)	0.223^***^ (0.031)	0.194^***^ (0.037)
December	0.378^***^ (0.042)	0.445^***^ (0.047)	0.402^***^ (0.035)	0.339^***^ (0.031)	0.343^***^ (0.033)	0.314^***^ (0.040)
Northwest	− 0.041^***^ (0.002)	− 0.079^*^ (0.047)	− 0.038 (0.035)	0.030 (0.031)	− 0.007 (0.033)	0.003 (0.039)
Yorkshire and Humber	− 0.085^***^ (0.001)	− 0.137^***^ (0.049)	− 0.095^***^ (0.036)	− 0.033 (0.032)	− 0.041 (0.034)	− 0.025 (0.041)
East Midlands	− 0.150^***^ (0.002)	− 0.214^***^ (0.050)	− 0.181^***^ (0.037)	− 0.124^***^ (0.033)	− 0.072^**^ (0.035)	− 0.048 (0.042)
West Midlands	− 0.166^***^ (0.002)	− 0.284^***^ (0.049)	− 0.155^***^ (0.036)	− 0.133^***^ (0.032)	− 0.078^**^ (0.034)	− 0.051 (0.041)
Eastern	− 0.302^***^ (0.004)	− 0.404^***^ (0.049)	− 0.329^***^ (0.036)	− 0.243^***^ (0.032)	− 0.175^***^ (0.034)	− 0.122^***^ (0.041)
London	− 0.351^***^ (0.004)	− 0.465^***^ (0.050)	− 0.396^***^ (0.037)	− 0.283^***^ (0.032)	− 0.226^***^ (0.035)	− 0.192^***^ (0.042)
Southeast	− 0.297^***^ (0.005)	− 0.396^***^ (0.046)	− 0.336^***^ (0.034)	− 0.233^***^ (0.030)	− 0.178^***^ (0.032)	− 0.113^***^ (0.038)
Southwest	− 0.216^***^ (0.003)	− 0.275^***^ (0.048)	− 0.237^***^ (0.036)	− 0.159^***^ (0.032)	− 0.155^***^ (0.034)	− 0.130^***^ (0.040)
Wales	− 0.085^***^ (0.001)	− 0.123^**^ (0.055)	− 0.120^***^ (0.041)	− 0.065^*^ (0.036)	− 0.034 (0.038)	− 0.029 (0.046)
Scotland	− 0.115^***^ (0.002)	− 0.139^***^ (0.049)	− 0.128^***^ (0.037)	− 0.074^**^ (0.032)	− 0.056 (0.034)	− 0.043 (0.041)
Northern Ireland	− 0.193^***^ (0.004)	− 0.264^***^ (0.052)	− 0.219^***^ (0.039)	− 0.117^***^ (0.034)	− 0.142^***^ (0.036)	− 0.105^**^ (0.043)
Linear monthly time trend	− 0.001^***^ (0.000)	− 0.002^***^ (0.000)	− 0.001^***^ (0.000)	− 0.001^***^ (0.000)	− 0.001^***^ (0.000)	− 0.001^***^ (0.000)
Constant	3.815^***^ (0.320)	3.909^***^ (0.598)	3.655^***^ (0.445)	3.720^***^ (0.389)	3.970^***^ (0.417)	4.146^***^ (0.500)
Observations	54,069	54,069	54,069	54,069	54,069	54,069

Significance: *0.1 **0.05 ***0.01. Standard errors in parentheses

## Footnotes

1 Moderate drinkers in this context are males (females) who drink more than 21 (14) units per week. Harmful drinkers are males (females) who drink more than 50 (35) units per week. A unit is equal to 10 ml or 8 g of pure alcohol.

2 Moreover, since this estimate comes from differencing across the pseudo-lifecycles of cohorts of individuals the interpretation that should be given to the resulting elasticity is that it is the response to anticipated variation in prices across the lifecycle. The policy elasticity of interest here is the conventional Marshallian one that tells us the response to unanticipated changes in price.

3 Censored quantile regression, which assumes all zero expenditures arise due to price/income reasons in a similar manner to the Tobit model, produce broadly similar results.

4 An alternative, where the regional element of the price index is removed to generate a single price index for the United Kingdom, is tested and reported in the “Appendix”. The results are broadly similar.

5 The finding might be interesting when looking at the shift from on-premise to off-premise consumption. A shift to the off-trade may change the probability of drink-driving or alcohol-related violence.

6 Our demand specification satisfies the conditions for exact aggregation, and implies constant elasticities. This implies that we would get the same estimated elasticities if we weighted the individual data together using any arbitrary weights.
